# Possum—A Framework for Three-Dimensional Reconstruction of Brain Images from Serial Sections

**DOI:** 10.1007/s12021-015-9286-1

**Published:** 2015-12-21

**Authors:** Piotr Majka, Daniel K. Wójcik

**Affiliations:** Nencki Institute of Experimental Biology, 3 Pasteur Street, 02-093 Warsaw, Poland; Department of Physiology, Monash University, Clayton, Victoria 3800 Australia

**Keywords:** 3D reconstruction, Histology, Image analysis, Image registration, Light microscopy, Brain atlas

## Abstract

Techniques based on imaging serial sections of brain tissue provide insight into brain structure and function. However, to compare or combine them with results from three dimensional imaging methods, reconstruction into a volumetric form is required. Currently, there are no tools for performing such a task in a streamlined way. Here we propose the Possum volumetric reconstruction framework which provides a selection of 2D to 3D image reconstruction routines allowing one to build workflows tailored to one’s specific requirements. The main components include routines for reconstruction with or without using external reference and solutions for typical issues encountered during the reconstruction process, such as propagation of the registration errors due to distorted sections. We validate the implementation using synthetic datasets and actual experimental imaging data derived from publicly available resources. We also evaluate efficiency of a subset of the algorithms implemented. The Possum framework is distributed under MIT license and it provides researchers with a possibility of building reconstruction workflows from existing components, without the need for low-level implementation. As a consequence, it also facilitates sharing and data exchange between researchers and laboratories.

## Introduction

In modern neuroscience we use multiple imaging techniques to study brain structure and function. The usage of complementary approaches is considered indispensable in characterizing the spatial organization of neuronal structures and their circuitry (e.g. Annese 2012; Leergaard et al. 2012; Osten and Margrie 2013). Despite exciting developments in brain imaging there is still no perfect technique which would allow for a comprehensive insight into all aspects of the brain over a wide range of spatial and temporal scales. From a practical point of view this means that data from non-destructive and destructive imaging methods have to be integrated.

The non-destructive techniques are methods to evaluate the properties of a brain without causing damage in a way allowing further experimental procedures on a specimen. The most common examples are different varieties of magnetic resonance imaging (MRI) or diffusion tensor imaging (DTI). These techniques allow one to obtain inherently three-dimensional (3D), virtually undistorted images, which deliver quantitative information on macroscopic tissue properties (e.g. Johnson et al. 2012), and can be applied *in vivo*.

Such techniques are commonly used to quantitatively characterize the morphology of brain structures (e.g. Badea et al. 2007; Herculano-Houzel et al. 2008), and their variation in populations (Ma et al. 2005), disease models (Sawiak et al. 2009), or behavioral studies (Lerch et al. 2011). Continuing improvement in spatial resolution of structural MR images (e.g. Johnson et al. 2007; Ullmann et al. 2012) made them methods of choice for deriving spatial references for multimodal brain atlases (e.g. Johnson et al. 2010; Papp et al. 2014; Hashikawa et al. 2015), including connectivity atlases (Veraart et al. 2011; Jiang and Johnson 2011).

Despite their versatility, such techniques suffer from several drawbacks. The most important are lower imaging resolution and lower specificity in comparison with methods based on microscopic imaging of sections stained with various techniques or injected with tracers.

The destructive techniques of brain imaging are methods which once applied prevent most further experimental procedures. They rely on obtaining series of two-dimensional images of brain tissue while sectioning or once the sections are cut and mounted on slides. Due to their specificity and microscopic scale of imaging they facilitate studies of fine properties of brain tissue at the cellular and sub-cellular levels. Typical examples are description of cyto- and chemoarchitecture of the neural tissue (e.g. Zilles 1985; Hof and Sherwood 2005) or axonal-level connectivity (e.g. Rosa et al. 2009; Zaborszky et al. 2015) via injections of different tracers. Other examples include autoradiography (e.g. Hess et al. 1998; Lebenberg et al. 2011), imaging unstained sections (Palm et al. 2010; Annese et al. 2014), or imaging sections which underwent *in-situ* hybridization process (Jagalur et al. 2007; Morris et al. 2010).

The capabilities of the 2D sectioning techniques have been elevated in recent years by high-throughput processing of sections and imaging methods (e.g. Chung et al. 2011; Ragan et al. 2012; Osten and Margrie 2013) along with relevant computational routines. This made it possible to conduct large-scale projects on gene expression (Lein et al. 2007), connectivity (Oh et al. 2014), and relating brain changes to behavior (Vousden et al. 2014; Kim et al. 2015).

The main drawback of sectioning approach is the necessity of physical dissecting of the brain which means it looses its three-dimensional integrity (e.g. cannot be sliced in a different plane). This is an undesirable side effect, since the brain is three dimensional and the imaging data should also be analyzed in a three-dimensional context. Additionally, comparison of section-based imaging data with results of 3D imaging methods requires the former to be brought into volumetric form (e.g. Dauguet et al. 2009; Johnson et al. 2010) which calls for adequate computational methods. Therefore, the integration of stacks of 2D images of stained sections into volumetric form is an indispensable aspect of modern neuroimaging and relevant methods and software for section alignment are actively developed.

The process of reconstruction of a series of 2D images into volumetric form is considered a difficult and time consuming task due to tissue distortions introduced during processing of the experimental material (Breen et al. 2005; Dauguet et al. 2007). These typically include global shrinkage and dehydration due to fixation in formaldehyde, freezing or paraffin embedding. Moreover, during cryosectioning, mounting and staining, additional distortions are incurred. Shearing, tearing and displacement of individual parts of the sections, non-uniform shrinkage due to chemicals used during staining procedures are common artifacts. Workflows aiming to faithfully reconstruct the 3D image of the brain have to account for such distortions (Qiu et al. 2011).

In its simplest form, the reconstruction can be performed by sequentially aligning consecutive sections to a reference section, usually the middle one (Kiessling 2011, p. 329). However, this approach is known to be volatile and sensitive to alignment imperfections and sections’ distortions (e.g. Nikou 2003). Moreover, the reconstructions performed this way tend to deviate from the true, anatomical shape. This phenomenon was nicknamed *the banana effect* (Malandain et al. 2004) and can be overcome by introducing a shape prior—a reference 3D image, which can be an MR image, collection of images of the face of the cutting block, atlas delineations, or a set of fiducial markers, which enforce the anatomical shape of the reconstruction.

Among the many reconstructions attempted, the recent work on the whole human brain stands out (Amunts et al. 2013; Annese et al. 2014). In both cases photographs of the face of the cutting block were taken while cryosectioning the brain. Individual sections were stained for cell bodies and then affinely aligned to corresponding blockface photographs which allowed to recover the anatomical brain shape. Additionally, the sections were nonlinearly corrected for section specific distortions which produced reconstruction of higher quality and made further coregistration to an MR image easier.

Adler et al. (2014) conducted a reconstruction of stained sections of human hippocampus using *post mortem* MR images as a shape prior. The process was first performed with multiple series of affine transformations followed by a correction of spatial artifacts performed by warping sections to their immediate neighbors and to the MR image simultaneously. Similarly, Chakravarty et al. (2006) performed a reconstruction of human basal ganglia and thalamus by applying affine alignment step followed by nonlinear corrections based on simultaneous warping towards both immediate neighbors of a given section.

Similar, multi stage reconstruction strategies were applied in non-human primate projects. For instance, in Choe et al. (2011) myelin-stained sections of owl monkey brain were registered to two-dimensional blockface images using a combination of linear and nonlinear methods prior to linear and nonlinear registration of the blockface volume to the MR image. An analogous strategy was used by Dauguet et al. (2009) in order to create a 3D digital atlas of the thalamus based on a series of stained histological sections of a baboon brain.

In reconstructions of rodents’ and other small animals’ brains similar strategies are employed. Some reconstructions are performed with the help of blockface images to which corresponding histological sections are aligned affinely (e.g. MacKenzie-Graham et al. 2004; Bertrand and Nissanov 2008; Mailly et al. 2010). Other approaches, like alignment based on landmarks, are also used (e.g. Hess et al. 1998).

Looking through the literature it seems that the present methodology crystallized around multistage workflows involving some form of a shape prior as an intermediate modality to which, after preprocessing, images are aligned affinely. Afterwards, nonlinear corrections are applied to account for distortions of individual sections. The resulting volume is then registered to a reference template which is either histology- or MR-based. Such strategies were utilized in, for instance, Lein et al. (2007), Uberti et al. (2009), and Lebenberg et al. (2011).

While it may seem that the reconstruction of images of serial sections should be a routine and standardized procedure, this is not the case. The above mentioned projects had specific goals, relied on different data modalities, used various numbers of specimens, etc. Additionally, the majority of the described reconstructions were done using workflows tailored to the goals of a particular project and appropriate software was not released publicly. Therefore, there is a lack of generic tools for handling this type of three dimensional reconstruction from serial sections. The available image registration tools for both 3D and 2D images (e.g. Klein et al. 2010; Avants et al. 2011; Peng et al. 2011) as well as for series of images (Thévenaz et al. 1998; Ribes et al. 2010; Cardona et al. 2012; Wang et al. 2015) perform excellently in their respective domains but they are not sufficient for reconstructions based of the whole brain histology for higher animals, from rodents to human and non-human primates, in a way mentioned in the earlier part of the introduction. A good example of these difficulties can be seen e.g. in Fig. 5 of Wang et al. (2014). The software proposed therein as a solution, despite implementing several useful methods (Wang et al. 2015), does not incorporate a reference image in the reconstruction process. Therefore, the issue of reconstructing section cut in an arbitrary plane (e.g. oblique) cannot be addressed and the banana effect cannot be mitigated.

In this article we present the Possum volumetric reconstruction framework, which is open software providing building blocks for constructing computational pipelines for reconstruction of 3D images based on serial sections. The software was created by selecting frequently utilized components of such workflows including: 1) naive affine sequential reconstructions, 2) affine sequential reconstructions designed to counteract the propagation of the artifacts, 3) routines for reconstruction with the presence of a shape prior, 4) deformable reconstruction workflow intended to account for distortions of individual sections. All workflows were implemented assuming that slices are ordered and have a constant thickness.

In addition to implementing the routines, we also validate the software using synthetic datasets illustrating properties of the provided algorithms. Additionally, we test the framework against publicly available MR and histology-based datasets. The validation results are reproducible and available as a part of the framework.

The problem addressed by the proposed software is the reconstruction of 3D images from series of stained sections. There is no attempt to handle coregistration of multiple 3D images as such a task is successfully addressed by existing software for intensity-based (e.g. Klein et al. 2010; Avants et al. 2011) or landmark-based (e.g. Peng et al. 2011) 3D image registration software.

## Methods

The framework has been developed using multiple technologies and comprises a collection of fundamental workflows used to generate 3D reconstructions and accompanying image processing routines. The body of the framework was implemented in the Python programming language (http://www.python.org) while the examples are available as shell scripts for easy customization and interaction with Linux-based operating systems.

InsightToolkit (ITK, RRID:nif-0000-00319, Schroeder 2005, http://www.itk.org/)compiled with Python wrappings (WrapITK, Lehmann et al. 2006), Convert3d (Yushkevich et al. 2006b, http://www.itksnap.org/, RRID:nif-0000-00317) and ImageMagick (http://www.imagemagick.org/) packages cover basic image processing operations such as reslicing, resampling, various kinds of filtering, cropping, stacking 2D images into volumetric form, data type conversion, etc. They also handle operations performed on transformations (e.g. composition).

The Possum framework relies on the Advanced Normalization Tools (ANTS, RRID:nlx_75959, Avants et al. 2011) software for conducting image registration. The ANTS package is used to perform both affine (including rigid) and deformable types of registration. The latter are carried out with the symmetric image normalization (SyN) method of Avants et al. (2008). Three image similarity metrics are used in the framework: cross-correlation coefficient (CC, Avants et al. 2008), Mattes’ mutual information (MI, Mattes et al. 2001) and mean square intensity difference (MSQ, Schroeder 2005) defined as:

$$\text{MSQ}(A,B) = \frac{1}{N} \sum\limits_{i=1}^{N} (A_{i} - B_{i})^{2}, $$

where *A*_*i*_, *B*_*i*_ are the intensities of the *i*-th pixels of images *A* and *B*, and *N* is the number of pixels comprising the images.

The Neuroimaging Informatics Technology Initiative file format (NIfTI, http://nifti.nimh.nih.gov/nifti-1) was selected as the data format of choice. It is capable of holding spatial information about the image (origin, spacing, orientation, anatomical directions, etc.) and storing 2D and 3D images using multiple data types (e.g. 8 bit, 16 bit, double and 24 bit per pixel RGB images which are used in the framework).

All command line software is invoked from Python scripts using a set of wrappers which allows for trivial parallelization using GNU Parallel (Tange 2011). Workflows were designed for execution in the parallel mode on either multi-core machines or on a computational clusters under supervision of a resource manager like TORQUE (http://www.adaptivecomputing.com/products/open-source/torque/) or SLURM (https://computing.llnl.gov/linux/slurm/).

### Graph-Based Affine Sequential Alignment

Graph-based sequential alignment (Yushkevich et al. 2006a) is a procedure for 3D reconstruction intended to minimize accumulation of registration errors and to identify highly distorted sections which might disturb the reconstruction process.

The implementation provided in the Possum framework is presented in Algorithm 1. In the first step every image is affinely aligned to *𝜖* neighboring sections towards either end of the stack. The CC metric with values rescaled to 〈−1,0〉 (the lower the value the more similar are the images) between each coregistered pair is recorded. Afterwards, a weighted graph (**G**) is built with vertices (**V**) representing individual images and edges (**E**) representing weights based on similarity between the images. 
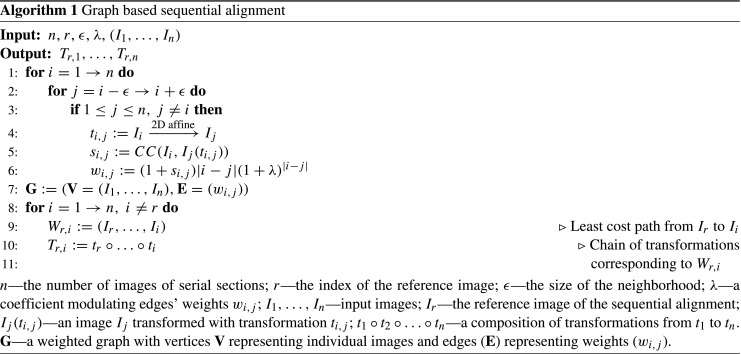


A reference section *I*_*r*_ is then selected, usually from the middle of the stack, and a transformation from *I*_*r*_ to any other image *I*_*i*_ is obtained by computing the least cost path *W*_*r*, *i*_ in the graph **G** using the Dijkstra algorithm (https://networkx.github.io/). This corresponds to a chain *T*_*r*, *i*_ of partial affine transformations *t*_*i*, *j*_ to be composed.

The number of chained transformations might be shorter than the nominal distance between the sections *I*_*r*_ and *I*_*i*_. This is interpreted as skipping those sections which are difficult to align to their neighbors. The preference to skip outstanding sections is adjusted with parameter *λ* which modulates the edges’ weights. Small positive *λ* favors section skipping while larger tends to preserve sections from being omitted in the transformation chain. Note that naive sequential alignment is a special case of this workflow for *𝜖* = 1 regardless of the *λ* value (Kiessling 2011).

### Iterative Affine Pairwise Alignment

The iterative affine pairwise alignment workflow allows one to construct a 3D image from a series of serial sections in the presence of a shape prior. It is particularly suitable for reconstructions in which the cutting plane of sections does not match the corresponding plane in the reference image, e.g. when coronal sections were cut at an angle with respect to the coronal plane defined in an atlas (Malandain et al. 2004; Yang et al. 2012; Adler et al. 2014).

This procedure simultaneously improves alignment of the reconstruction to the reference image by calculating a global 3D affine transformation between the reconstruction and the reference. At the same time it aligns the experimental sections to the corresponding virtual reference cuts (see Algorithm 2). Eventually, the algorithm converges to a reconstruction which is affinely aligned to the the reference image and in which the sections are broadly consistent between one another. 
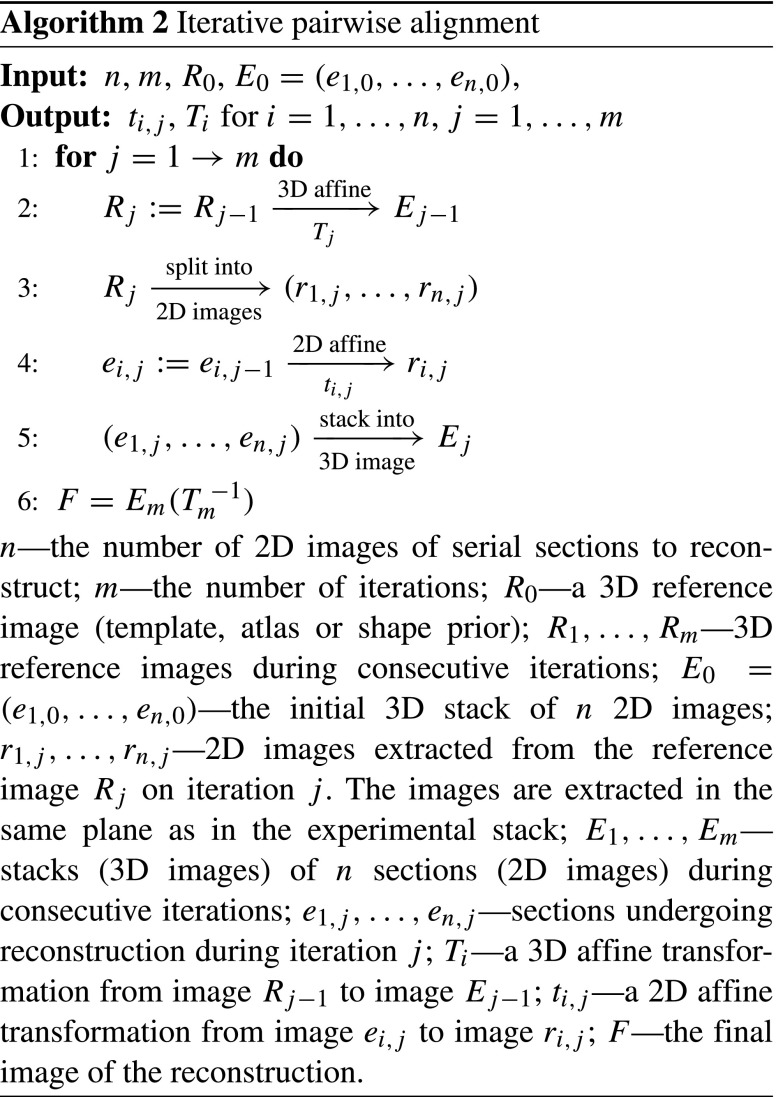


### Coarse-to-fine Alignment

The coarse-to-fine reconstruction approach proposed by Yushkevich et al. (2006a) uses a reference image (shape prior) in which each experimental section has a corresponding section from the reference image assigned. The method is intended to account for the accumulation of registration errors, a Z-shift (the banana effect), and to mitigate severe discontinuities in the reconstruction.

The overall idea of the approach is to perform and then combine two series of rigid registrations to produce a faithful reconstruction. The first, coarse-scale registration, relies on aligning images being reconstructed to corresponding sections of the reference image. Such sections can be obtained, for instance, by using the Iterative Affine Pairwise Alignment and then resampling the transformed reference image in the space defined by the stack of histological images. This series of transformations recovers the overall shape of the brain but does not yield accurate section-to-section alignment.

The second series of transformations, the fine-scale registration, is realized by any kind of sequential alignment workflow (e.g. naive or graph-based), and aims to provide pairs of neighboring sections well aligned to one another. A Z-shift might be introduced in this stage and the overall shape of the reconstruction might be different from the reference one.

To obtain the final result, the high-frequency component of the fine-scale alignment is combined with the coarse-scale registration. This is done by Gaussian smoothing of individual parameters of the fine-scale transformation (translation and rotation angle) across the z (stack) dimension and filtering them out before combining with the parameters of the coarse-scale registration. This yields a reconstruction which preserves both the global shape of the brain and local anatomical details, combining advantages of both coarse and fine registration.

### Deformable Reconstruction

The method for deformable refinement of the reconstruction of the histological volume stems from an assumption that a change of shape of a brain structure is slower than the section thickness. Thus the neighboring images are similar to one another in a formal sense (Chakravarty et al. 2006; Ju et al. 2006; Adler et al. 2014). Analysis of the theoretical properties of the method was provided by Gaffling et al. (2014).

The elementary step of the method consists of registration of the image of a given section *M*_*i*_ to a fixed image *F*_*i*_ obtained by averaging images of sections in *𝜖*-neighborhood of section *M*_*i*_. Such an alignment is performed for each image in the stack which constitutes a single iteration (Algorithm 3). An arbitrary number of iterations can be conducted and the deformable registration parameters may vary between consecutive iterations. 
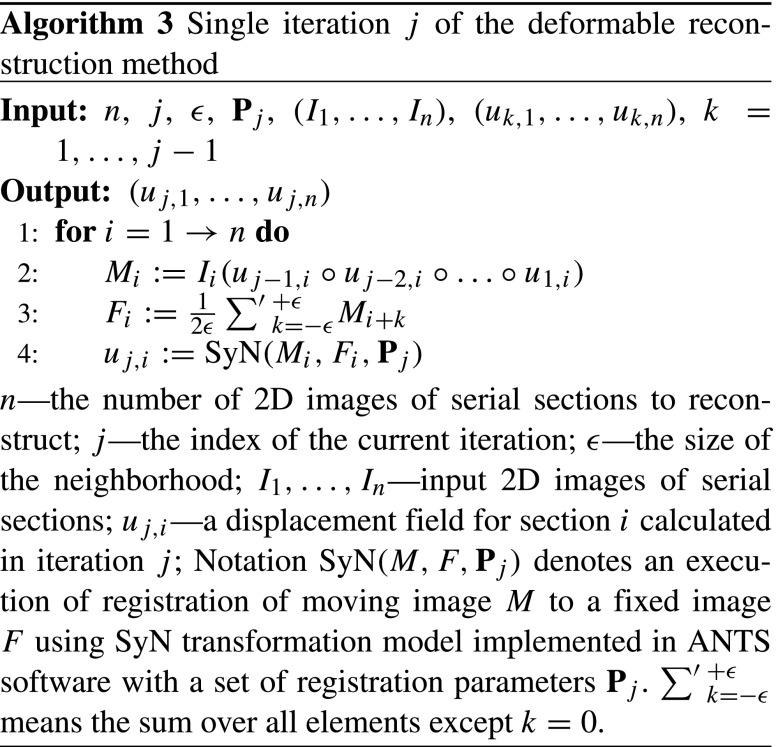


This process eliminates high frequency discontinuities caused by section-specific distortions effectively separating the anatomy from the deformation (Gaffling et al. 2014). This translates into smoother and easier to distinguish anatomical structures.

## Results

### Coarse-to-fine Workflow

**Fig. 1:**
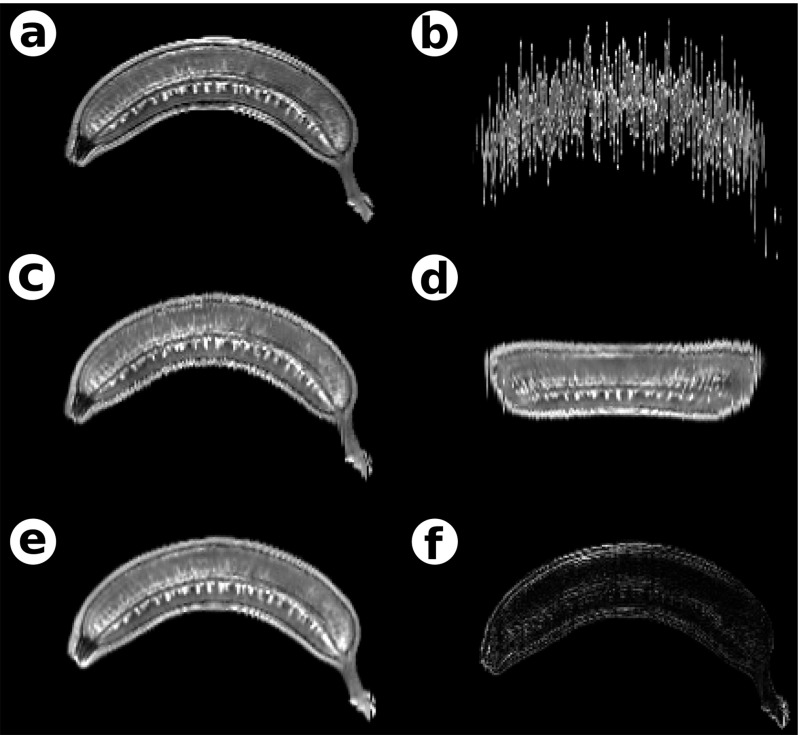
Results of the coarse-to-fine reconstruction workflow (Yushkevich et al. 2006a) applied to a synthetic dataset based on an MR image of the banana, sagittal cross-sections. **a** The initial, undistorted image, *I*_*S*_; **b** a distorted image; **c** a coarse-scale reconstruction obtained by aligning individual sections from panel b to corresponding sections from panel a, *I*_*C*_; **d** a fine-scale step—naive sequential alignment, *I*_*F*_; deviation from the true shape is clearly visible; **e** the result of merging coarse- and fine-scale steps, *I*_*M*_; f) the discrepancy between images *I*_*S*_ and *I*_*M*_

To illustrate the properties of the coarse-to-fine reconstruction workflow, a training T2–weighted MR image with an isotropic voxel resolution of 1 mm ^3^ of a curved banana was used (*I*_*S*_, Fig. 1a). The image was distorted by randomly translating and rotating each of the 200 individual banana slices (Fig. 1b). The amount of translation was drawn from a Gaussian distribution with *μ* = 0 mm, *σ* = 10 mm for translation in each direction and *μ* = 0^∘^ and *σ* = 10^∘^ for rotation. Then, a coarse-to-fine reconstruction was performed during which the distorted image was reconstructed using the undistorted image *I*_*S*_ as a shape prior. In the coarse-scale step, the images were aligned rigidly to the corresponding section of the undistorted image, and MI similarity metric was used. The fine-scale transformation series was calculated using the naive sequential alignment, with 110 ^th^ section designated as the reference, rigid alignment and MI similarity metric. The transformation merge was performed with *σ* = 5 sections for both translation and rotation parameters of the rigid transformations.

The results of the coarse-scale reconstruction (*I*_*C*_, Fig. 1c) show that it recovered the true shape of the phantom, however, the neighboring sections are only roughly aligned to one another; MSQ(*I*_*S*_, *I*_*C*_) = 55. The fine-scale step (*I*_*F*_, Fig. 1d) resulted in reconstruction in which sections were well aligned to one another although with notable z-shift and volume twist; MSQ(*I*_*S*_, *I*_*C*_) = 663.

The merge of the two transformation series (*I*_*M*_, Fig. 1e) provided a reconstruction which preserves the true global shape and has a high section-to-section coherence; MSQ(*I*_*S*_, *I*_*M*_) = 39, (Fig. 1f).

### Graph-Based Affine Reconstruction

**Fig. 2:**
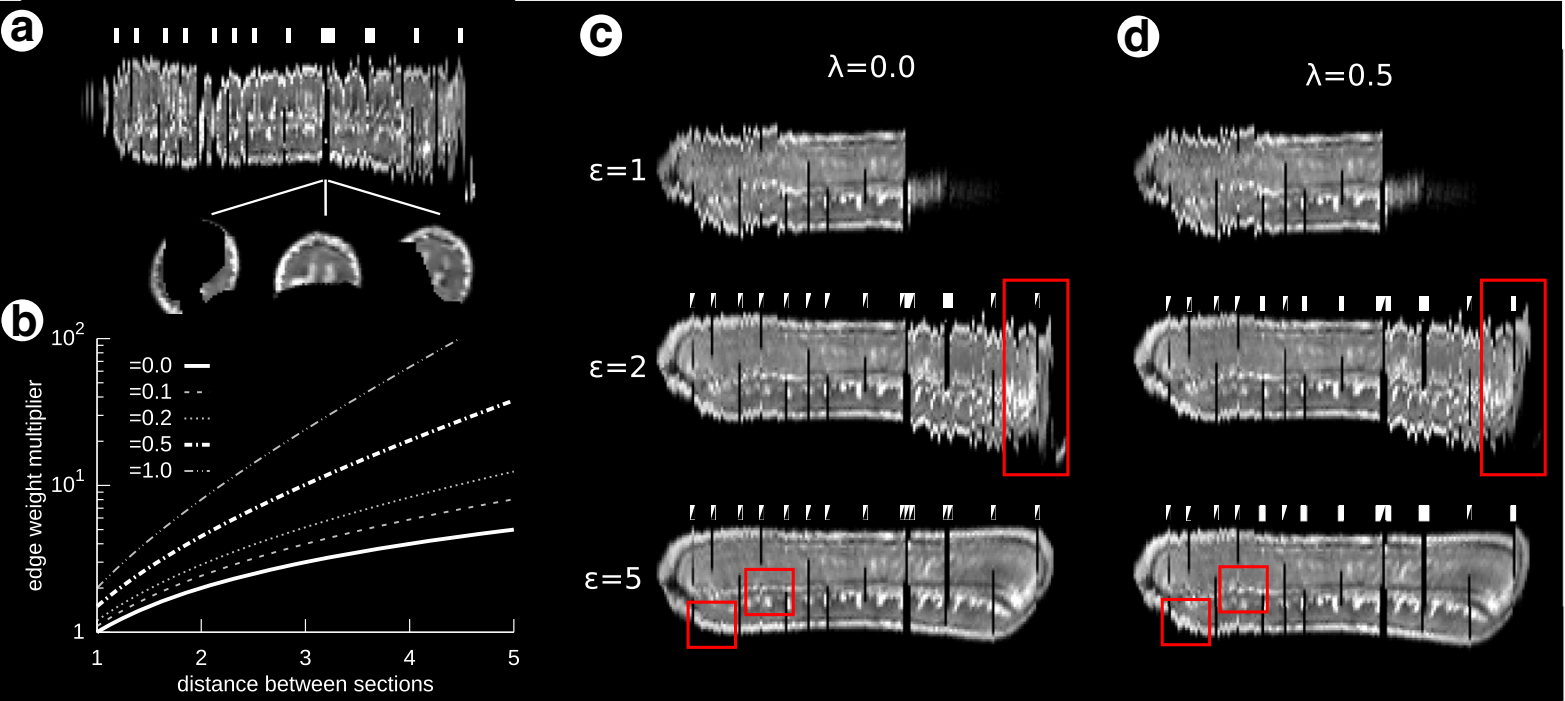
Assessment of the graph-based sequential alignment. **a** A sagittal cross-section of the distorted MR image of the banana. *White rectangular markers* indicate sections in which some parts were removed to introduce severe distortion; 15 sections were prepared in this way including groups of three and two consecutive distorted sections. Example sections with severe distortions are shown below the cross section. **b** Graph edge weight multiplier depending on distance between sections and value of *λ* parameter according to Algorithm 1, line 6; **c**, **d** Cross sections of the reconstructions performed with different *λ* and *𝜖* parameters. *White rectangular markers* show heavily distorted sections which have not been omitted while *wedges* indicate sections successfully recognized as standing out

An MR image of the naive-sequentially aligned banana (Fig. 1d) was used to prepare a synthetic dataset to assess the efficiency of the graph-based sequential alignment. The image was distorted by applying randomly generated affine transformations independently to each section (Fig. 2a). Additional distortions were introduced by manually removing approximately a half of the banana slice on 15 sections. In particular, groups of three and then two successive distorted sections were created in this way.

The distorted image underwent the graph-based sequential alignment with various reconstruction settings. Tested values of the neighborhood radius were *𝜖* = 1,2,5 and *λ* values of 0 and 0.5 were used (Fig. 2b).

The results of the reconstruction (Fig. 2c, d) depend on values of both parameters, *𝜖* and *λ*. The primary difference is the amount of the recovered shape for different values of the *𝜖* parameter. For *𝜖* = 1, which is equivalent to the naive sequential alignment, highly distorted sections cannot be omitted regardless of the lambda value (see *𝜖* = 1 in Fig. 2c, d). This is enhanced when several heavily distorted sections follow in a row.

Increasing the neighbor size to 2 makes it possible to handle two successive distorted sections. Consequently, further increment of the *𝜖* value allows the method to detect and omit more distorted sections for both tested *λ* values (*𝜖* = 2,3 in Fig. 2c, d). Comparing the reconstructions performed with different *λ* values confirms that the higher the *λ* the fewer distorted sections are skipped which is in accordance with the assumptions of the method and curves shown in Fig. 2b.

### Deformable reconstruction

The dataset used to illustrate the deformable reconstruction routine was a T2*-weighted MR image of an 80 days old Wistar rat brain (Johnson et al. 2012). The original image was downsampled to 25×50×50 *μm*^3^ voxel size which corresponds to 1600×400×400 (coronal, sagittal, horizontal planes, accordingly) voxels. The image was sliced in the coronal plane and the in-plane resolution of 50×50 *μm*^2^ was preserved while the thickness of the synthetic coronal sections, *d*, ranged from 20 to 100 *μ*m in the intervals of 5 *μ*m in different reconstruction trials. The reference images obtained with this procedure for a given section thickness *d* will further be denoted by *R*_*d*_.

Subsequently, the synthetic coronal sections were nonlinearly distorted to mimic deformations naturally occurring during the preparation of the histological sections. The distortions were modeled by an application of a 2D displacement field *T*_*σ*_, with *σ* characterizing spatial correlations. Each component of the displacement field was constructed from a 2D white noise image smoothed with a Gaussian filter with kernel size of *σ* = 300 *μ*m rescaled to 〈−*r*, *r*〉. The deformation amplitude *r* was chosen so as to set the median magnitude of the displacement vector 〈*T*_*σ*_〉=50 *μ*m which is a value obtained (Majka 2014, p. 47). The procedure was repeated for all sections in the stack yielding a 3D image with individually distorted coronal sections denoted by *D*_*d*_. Such an image underwent the procedure of deformable reconstruction.

We used the ANTS software to perform registration between individual images in the deformable reconstruction process using the following procedures: SyN transformation model with the gradient step of 0.025 and CC similarity metric with a kernel size of 2 voxels; Gaussian regularization with a sigma of 1 voxel for both similarity gradient and displacement field; five level multi-resolution registration scheme with 1000 iterations at each level. These settings remained unchanged for all trials. Each reconstruction trial consisted of 20 iterations. The neighborhood parameters were *𝜖* = 1,2,…,10, and the synthetic coronal section thickness was *d* = 20,25…,100 *μ*m. For each pair of parameters (*𝜖*, *d*) three reconstruction trials were conducted which amounted to 510 trials. Reconstructed image after iteration *i* corresponding to distorted image *D*_*d*_ is denoted as *B*_*d*, *𝜖*, *i*_.

Next, we studied the reconstruction accuracy. To determine how close the reconstructions were to the initial image, for each reconstruction trial we computed the MSQ similarity measure between the reference image *R*_*d*_, the distorted image *D*_*d*_, and the reconstructed image *B*_*d*, *𝜖*, *i*_.

In the first step we determined the values of *𝜖* for which the reconstructions were most similar to the original. For a tuple of three parameters (*d*, *𝜖*, *i*) the value of *𝜖* was chosen so that

$$\epsilon^{\ast} = \underset{\epsilon}{\arg\min}\; \text{MSQ}(R_{d}, B_{d,\epsilon,i})|_{d,i}. $$

In 46 cases out of 51 the most accurate reconstructions were achieved for *𝜖*^∗^ = 1, in the remaining 5 cases it was *𝜖*^∗^ = 2. We set *𝜖* = 1 for further analyses.

In the next step the number of iterations yielding optimal reconstruction was identified by choosing the iteration index *i*^∗^ leading to the highest improvement in comparison with the initial distorted image:

1 $$\begin{array}{@{}rcl@{}} i^{\ast} &=& \underset{i}{\arg\min}\; \left\langle S_{d,\epsilon=1,i} \right\rangle \\ & = &\underset{i}{\arg\min}\; \left\langle \frac{\text{MSQ}(R_{d}, B_{d,\epsilon=1,i}) }{ \text{MSQ}(R_{d}, D_{d})} \right\rangle_{\text{3 trials}}. \end{array} $$

We will refer to the measure used here as relative similarity.

**Fig. 3:**
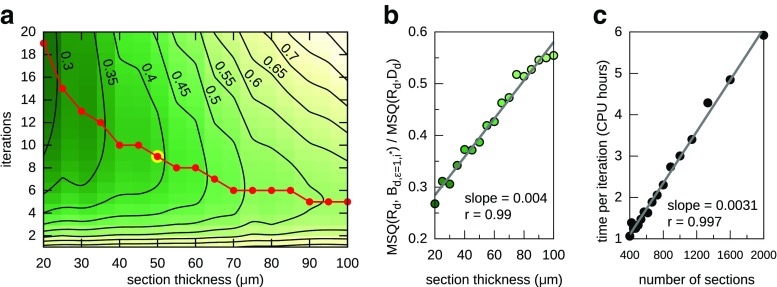
Results of the deformable reconstruction workflow assessment. **a** A heatmap showing the accuracy of the reconstruction (1) depending on the thickness of the synthetic coronal sections and the number of iterations in the trial. The *red points* and *accompanying line* indicate the number of iterations for which, on average, the reconstruction was the most accurate given the section thickness (*i* ^∗^, see Eq. 1). The reconstruction trial marked with the *yellow point* corresponds to the case shown in Fig. 4. **b** Values of the relative similarity of the most accurate reconstruction trials depending on the section thickness. **c** The total processing time per iteration (CPU hours) elapsed on the reconstruction trials depending on the number of sections

The results show that the thinner the section is (the larger the number of sections) the larger number of iterations is required to reach the most accurate reconstruction for the given section thickness (Fig. 3a). For the 20 *μ*m sections the optimal number of iterations was 19 and it systematically lowered with increasing thickness so that for the section thickness of 100 *μ*m (400 sections) only 5 iterations were required to get the reconstruction most similar to the undistorted image. Note that when the number of iterations grows, the reconstruction diverges from the reference image, thus one needs additional measures to identify the optimal reconstruction. In practical applications, when the undistorted image is unknown, the stop-point can be determined, for instance, either by letting a neuroanatomist to decide which reconstruction is the most satisfactory or by tracking the changes (e.g. calculating the MSQ value) between consecutive reconstructions and terminating the process once the difference reaches an arbitrarily defined value or the first local minimum.

Additionally, the reconstruction accuracy lowered as the section thickness increased. This is expressed by the values of the relative similarity which grew linearly with the section thickness (Fig. 3b). For the initial 20 *μ*m thickness it was 0.27 and increased up to 0.55 for the 100 *μ*m sections with the factor of 4⋅10^−4^ per *μ*m.

To assess how the deformable reconstruction workflow scales up with the different number of sections being reconstructed, the total CPU time elapsed on each reconstruction trial (consisting of twenty iterations) was recorded (Fig. 3c). The time increased from 21.2 CPU hours (1.1 hour per iteration) for 400 sections (section thickness of 100 *μ*m) to 118 h (5.9 h per iteration) for 2000 sections (20 *μ*m thick). On average, the time required to conduct a single reconstruction trial increased with a factor of 0.0031 CPU hours (11.36 s) per section per iteration. The tests were performed under Ubuntu 10.04 operating system deployed on a dual Intel®; Xeon®; E5620 (16×2.40 GHz logical processors) server equipped with 32 GB of RAM.

**Fig. 4:**
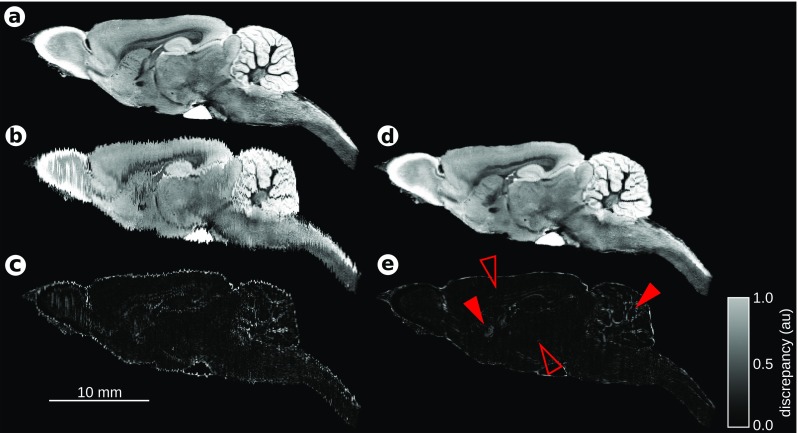
Sagittal cross sections of a single trial in the assessment of the deformable reconstruction workflow during consecutive stages of processing. **a** The reference image *R*_50*μm*_, **b** a distorted image *D*_50*μm*_, **c** the difference between images *a* and *b* showing high discrepancy close to the brain outline, inside the cerebellum, olfactory bulb, and striatum, **d** a reconstructed image $B_{d=50~\mu m, \epsilon =1,i^{\ast }=9}$, **e** the difference between images presented on panels *a* and *d* showing that the discrepancy was significantly reduced after conducting the reconstruction process. *Empty triangles* denote regions in which the reconstruction was successful while *filled triangles* indicate worse performance

An example reconstruction conducted for the section thickness of 50 *μ*m, *𝜖* = 1 and *i*^∗^ = 9 (yellow point in Fig. 3a) was selected for presentation in Fig. 4. We can see that the reconstruction was the most accurate in regions where the brain structure changed the least and for structures of low curvature, e.g. neocortex, thalamus, hypothalamus, midbrain, pons (empty triangles in Fig. 4e). On the other hand, structures with complex internal details and substantial curvature, e.g. cerebellar cortex, striatum, suffered from some reconstruction artifacts like segment-wise overstraightening and general difficulty in recovering fine details of curvature (filled triangles in Fig. 4e). Some of the artifacts are noticeable also in the olfactory bulb.

### Evaluation on an Open Histological Dataset

To demonstrate the capabilities of the framework we performed a reconstruction based on the Waxholm Space Mouse Brain Atlas (Johnson et al. 2010). The dataset included (21.5 *μ*m) ^3^ isotropic T2*-weighted MR image of the 80 days old CJ57BL/6 mouse brain and a series of 312 images of horizontal Nissl-stained sections of the same brain. To streamline the calculations and make the data convenient to share as an example, the original MR image was downsampled to isotropic (43 *μ*m) ^3^ resolution and the high-resolution Nissl-stained sections were downsampled to pixel size of 50×50 *μm*^2^. A reconstruction workflow comprising the following four steps was applied.

To begin with, ten iterations of the pairwise registration workflow were conducted. During this step the histological images were rigidly aligned to the corresponding virtual sections obtained by affinely aligning the reference image to the stack of histological images being reconstructed. Correlation coefficient (CC) was used as the image similarity metric. This stage resulted in a rough registration of the histological image stack to the reference MR image.

Subsequently, graph-based sequential alignment with *𝜖* = 5 and *λ* = 0 was applied to the results of the previous step. The image of the 110 ^th^ section was used as the reference to which all the remaining section were aligned sequentially using rigid transformations and MI as the image similarity metric.

The last step was to apply eight iterations of the deformable reconstruction workflow. During this process the following parameters were used: *𝜖* = 1; CC image similarity metric with the kernel size of 2 voxels; gradient step of 0.01; Gaussian regularization with kernel size of 100 *μ*m for similarity gradient and 50 *μ*m for the displacement field; six-level image pyramid with 1000 iterations per level.

**Fig. 5:**
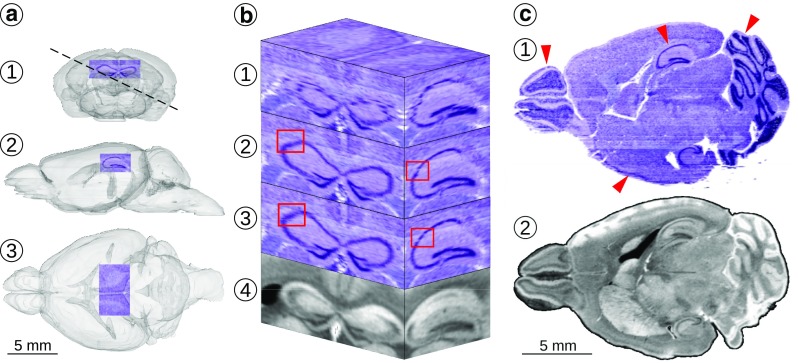
Results of a 3D reconstruction of the Nissl-stained sections (Johnson et al. 2010). Column **a**: the outline of the 3D reconstruction of the brain (*gray model*) and the location of the cuboid shown in the column (**b**). Coronal (*a1*), lateral (*a2*), and horizontal (*a3*) projections. The plane shown in panel (*a1*) was used to obtain oblique cuts through the reconstruction and the reference image presented in column (**c**). Column **b**: Fragments of the reconstructions showing dentate gyrus and neighboring structures during consecutive stages of processing, 1) pairwise alignment, 2) graph-based sequential alignment, 3) deformable reconstruction, the final stage, 4) the reference MR image. Column **c**: Oblique cuts through the reconstructions: 1) the final reconstruction, 2) the reference MR image. *Red arrows* on panel *c1* indicate successful, detailed reconstructions of olfactory bulb, piriform cortex, hippocampus, and cerebellum

Ultimately, transformations from all the intermediate steps for every section were merged and used to reslice this section. The reconstruction yielded 3D brain image coregistered affinely to the reference MR image (Fig. 5). Additionally, the same transformations were applied to the masks of sections which provided a reconstruction of the brain outline (Fig. 5a).

## Discussion and Summary

In this article we introduced the Possum framework, software addressing the task of reconstruction of three-dimensional images of the brain based on series of two-dimensional images of stained sections. To develop the framework we reviewed and selected workflows which accomplish versatile reconstruction tasks according to today’s best practices. Additionally, we demonstrated properties of individual routines and illustrated which reconstruction task they are suitable for.

The framework design paradigm was to utilize reliable open source components and follow guidelines for designing and evaluating scientific software (e.g. Tustison et al. 2013). This decision resulted in both increase of the framework’s stability and reduction of the development efforts. The primary external component is the ANTS software, chosen because it is a thoroughly tested (Klein et al. 2009), customizable, and task-agnostic image registration tool. By using open source and well maintained image processing libraries (e.g. ITK) and NIfTI file format which are *de facto* standards (Poline et al. 2012; Avants et al. 2015) in neuroimaging we increased the interoperability between the framework and other pre- and post-processing tools. However, the chosen paradigm caused an overhead due to data exchange between individual components and resulted in hampering, to some extent, the efficiency of the framework. Replacing external dependencies with dedicated components is a part of undergoing maintenance work. The scalability of the software is mainly a result of enabling parallel processing without which handling sizable datasets would be inconvenient. Due to the nature of the data—multiple images which usually can be processed independently—naive parallelization turned out to be a sufficient solution. To assess the scalability we tested the framework against both, relatively small synthetic datasets of the banana as well as large ones, containing up to two thousand sections. The framework managed to handle both situations well.

The implemented workflows constitute a software collection which allows one to conduct typical tasks of reconstructing 3D brain images from series of stained sections assuming they are properly ordered and of constant thickness. Graph-based affine sequential alignment and coarse-to-fine routines allow one to conduct affine reconstructions with or without using a shape prior while at the same time reducing Z-shift, skewing, banana effect, and propagation of the alignment errors due to the sections’ distortions. The iterative alignment workflow makes it possible to perform the reconstruction within the coordinate space of the reference image accounting for the fact that the sections were cut in a different plane than the corresponding sections in the reference image. The deformable reconstruction workflow uses nonlinear transformations to compensate for section-specific distortions, eliminating discontinuities, improving overall reconstruction quality, and making it easier to conduct further 3D to 3D coregistration tasks. Individual reconstruction routines can be stacked to create pipelines tailored to specific projects.

With the example of Nissl stained sections of the Waxholm Space Mouse Brain we showed that the framework is capable of tackling data from demanding research projects. The workflow used there addresses a typical reconstruction task in which a 3D brain image is reconstructed from a series of stained sections and a reference image. Additionally, during its development, the framework was used to create stereotaxic atlas of the *Monodelphis* opossum brain (Majka et al. 2013) and to construct a workflow for connectivity data mapping in the common marmoset brain (Majka et al. 2014).

One aspect of 3D reconstruction not addressed by methods presented in this article is repairing highly distorted sections (e.g. detached parts of the tissue, tears, etc.). Different approaches have been utilized to mitigate such distortions. For instance, Choe et al. (2011) corrected displaced parts of sections by identifying them manually in, both, histological and reference (blockface) images. A more automated approach was proposed by Dauguet et al. (2007) where semi-automatic hemisphere separation was performed assuming that the processed sections were symmetric. A more elaborate approach was used by Amunts et al. (2013), who iteratively perform 3D reconstruction and remove minor artifacts while severe distortions are still identified and corrected manually.

For detached and torn pieces of tissue methods similar to those proposed by Pitiot et al. (2006) and Pitiot and Guimond (2008) seem to be a good remedy. Briefly, their algorithms model such distortions with several rigid or affine local transformations embedded in an elastic one. Not only it allows to automate repairing heavily distorted sections but also makes it possible to encode such corrections as displacement fields which is important from the reproducibility standpoint.

### Framework in the Context of Digital Brain Atlasing

The presented software addresses the key issue of creating 3D images from serial sections before deformable mapping to a three-dimensional reference space using existing software. By reducing the efforts of establishing 3D reconstruction pipeline and providing reliable routines, the Possum framework gives the researchers an opportunity to conduct projects involving histological data integration by themselves. The framework might be used for instance to facilitate delineation of brain structures based on both, MR images and histology (e.g. Kumazawa-Manita et al. 2013; Ullmann et al. 2015), or in brain connectivity studies (Kuan et al. 2015; Sukhinin et al. 2015).

Another example might be processing legacy data by which we understand histological experimental material which has been collected without intention to reconstruct in it 3D but which may still constitute a valuable neuroscientific resource and therefore would benefit from integration with other digital atlasing resources, such as Zakiewicz et al. (2015).

### Further Directions and Outlook

The directions of further development are twofold. In terms of the framework functionality, the next step is to provide routines for preprocessing of the input data, e.g. interfaces for managing collections of input images, implementing routines for correcting section staining inhomogeneities (e.g. Chakravarty et al. 2006; Yelnik et al. 2007; Ceritoglu et al. 2010), fixing tears or severe displacements of the tissue (e.g. Dauguet et al. 2007; Pitiot and Guimond 2008) before conducting the actual reconstruction process. With regard to the framework architecture, the next step is to develop a mechanism for easy connecting, interfacing between consecutive steps and monitoring execution of the pipeline (e.g. Gorgolewski et al. 2011; Friedel et al. 2014). We ultimately envision the framework as a back-end of a web service connected to high resolution image repositories (e.g. Mikula et al. 2007) which would allow one to compose and execute various reconstruction pipelines.

## Information Sharing Statement

The source code of the framework is distributed under the terms of the MIT license and is available to download from the GitHub repository: https://github.com/pmajka/poSSum. The repository contains also the code and data necessary to reproduce examples shown in this article.

A preconfigured virtual machine with ready-to-use installation of the framework as well as the code used to perform the reconstruction of the Waxholm Space Mouse Brain Reference can be found at: http://www.3dbar.org/wiki/barPosSupp.

## References

[CR1] Adler DH, Pluta J, Kadivar S, Craige C, Gee JC, Avants BB, Yushkevich PA (2014). Histology-derived volumetric annotation of the human hippocampal subfields in postmortem MRI. NeuroImage.

[CR2] Amunts K, Lepage C, Borgeat L, Mohlberg H, Dickscheid T, Rousseau M-E, Bludau S, Bazin P-L, Lewis LB, Oros-Peusquens A-M, Shah NJ, Lippert T, Zilles K, Evans AC (2013). BigBrain: an ultrahigh-resolution 3D human brain model. Science (New York, N.Y.).

[CR3] Annese J (2012). The importance of combining MRI and large-scale digital histology in neuroimaging studies of brain connectivity and disease. Frontiers in Neuroinformatics.

[CR4] Annese J, Schenker-Ahmed NM, Bartsch H, Maechler P, Sheh C, Thomas N, Kayano J, Ghatan A, Bresler N, Frosch MP, Klaming R, Corkin S (2014). Postmortem examination of patient H.M.’s brain based on histological sectioning and digital 3D reconstruction. Nature Communications.

[CR5] Avants BB, Epstein CL, Grossman M, Gee JC (2008). Symmetric diffeomorphic image registration with cross-correlation: evaluating automated labeling of elderly and neurodegenerative brain. Medical Image Analysis.

[CR6] Avants BB, Johnson HJ, Tustison NJ (2015). Neuroinformatics and the The Insight ToolKit. Frontiers in Neuroinformatics.

[CR7] Avants BB, Tustison NJ, Song G, Cook PA, Klein A, Gee JC (2011). A reproducible evaluation of ANTs similarity metric performance in brain image registration. NeuroImage.

[CR8] Badea A, Ali-Sharief AA, Johnson GA (2007). Morphometric analysis of the C57BL/6J mouse brain. NeuroImage.

[CR9] Bertrand L, Nissanov J (2008). The Neuroterrain 3D Mouse Brain Atlas. Frontiers in Neuroinformatics.

[CR10] Breen MS, Lancaster TL, Wilson DL (2005). Correcting spatial distortion in histological images. Computerized Medical Imaging and Graphics: the Official Journal of the Computerized Medical Imaging Society.

[CR11] Cardona, A., Saalfeld, S., Schindelin, J., Arganda-Carreras, I., Preibisch, S., Longair, M., Tomancak, P., Hartenstein, V., & Douglas, R.J. (2012). TrakEM2 software for neural circuit reconstruction. *PLoS ONE*, *7* (6). 10.1371/journal.pone.0038011 PMC3378562 22723842

[CR12] Ceritoglu C, Wang L, Selemon LD, Csernansky JG, Miller MI, Ratnanather JT (2010). Large deformation diffeomorphic metric mapping registration of reconstructed 3D histological section images and in vivo MR images. Frontiers in Human Neuroscience.

[CR13] Chakravarty MM, Bertrand G, Hodge CP, Sadikot AF, Collins DL (2006). The creation of a brain atlas for image guided neurosurgery using serial histological data. NeuroImage.

[CR14] Choe AS, Gao Y, Li X, Compton KB, Stepniewska I, Anderson AW (2011). Accuracy of image registration between MRI and light microscopy in the ex vivo brain. Magnetic Resonance Imaging.

[CR15] Chung JR, Sung C, Mayerich D, Kwon J, Miller DE, Huffman T, Keyser J, Abbott LC, Choe Y (2011). Multiscale exploration of mouse brain microstructures using the knife-edge scanning microscope brain atlas. Frontiers in Neuroinformatics.

[CR16] Dauguet J, Condé F, Hantraye P, Frouin V, Delzescaux T (2009). Generation of a 3D atlas of the nuclear division of the thalamus based on histological sections of primate: Intra- and intersubject atlas-to-MRI warping. IRBM.

[CR17] Dauguet J, Delzescaux T, Condé F, Mangin J-F, Ayache N, Hantraye P, Frouin V (2007). Three-dimensional reconstruction of stained histological slices and 3D non-linear registration with in-vivo MRI for whole baboon brain. Journal of Neuroscience Methods.

[CR18] Friedel M, van Eede MC, Pipitone J, Chakravarty MM, Lerch JP (2014). Pydpiper: a flexible toolkit for constructing novel registration pipelines. Frontiers in Neuroinformatics.

[CR19] Gaffling S, Daum V, Steidl S, Maier A, Kostler H, Hornegger J (2014). A Gauss-Seidel iteration scheme for reference-free 3-D histological image reconstruction. IEEE Transactions on Medical Imaging.

[CR20] Gorgolewski, K., Burns, C.D., Madison, C., Clark, D., Halchenko, Y.O., Waskom, M.L., & Ghosh, S.S. (2011). Nipype: a flexible, lightweight and extensible neuroimaging data processing framework in Python. *Frontiers in Neuroinformatics*, *5*. 10.3389/fninf.2011.00013 PMC3159964 21897815

[CR21] Hashikawa T, Nakatomi R, Iriki A (2015). Current models of the marmoset brain. Neuroscience Research.

[CR22] Herculano-Houzel S, Collins CE, Wong P, Kaas JH, Lent R (2008). The basic nonuniformity of the cerebral cortex. Proceedings of the National Academy of Sciences of the United States of America.

[CR23] Hess A, Lohmann K, Gundelfinger ED, Scheich H (1998). A new method for reliable and efficient reconstruction of 3-dimensional images from autoradiographs of brain sections. Journal of Neuroscience Methods.

[CR24] Hof, P.R., & Sherwood, C.C. (2005). Morphomolecular neuronal phenotypes in the neocortex reflect phylogenetic relationships among certain mammalian orders. In *Anatomical Record - Part A Discoveries in Molecular, Cellular, and Evolutionary Biology*, (Vol. 287 pp. 1153–1163). 10.1002/ar.a.20252 16211636

[CR25] Jagalur M, Pal C, Learned-Miller E, Zoeller RT, Kulp D (2007). Analyzing in situ gene expression in the mouse brain with image registration, feature extraction and block clustering. BMC bioinformatics.

[CR26] Jiang Y, Johnson GA (2011). Microscopic diffusion tensor atlas of the mouse brain. NeuroImage.

[CR27] Johnson GA, Ali-Sharief A, Badea A, Brandenburg J, Cofer G, Fubara B, Gewalt S, Hedlund LW, Upchurch L (2007). High-throughput morphologic phenotyping of the mouse brain with magnetic resonance histology. NeuroImage.

[CR28] Johnson GA, Badea A, Brandenburg J, Cofer G, Fubara B, Liu S, Nissanov J (2010). Waxholm space: an image-based reference for coordinating mouse brain research. NeuroImage.

[CR29] Johnson GA, Calabrese E, Badea A, Paxinos G, Watson C (2012). A multidimensional magnetic resonance histology atlas of the Wistar rat brain. NeuroImage.

[CR30] Ju T, Warren J, Carson J, Bello M, Kakadiaris I, Chiu W, Thaller C, Eichele G (2006). 3D volume reconstruction of a mouse brain from histological sections using warp filtering. Journal of Neuroscience Methods.

[CR31] Kiessling F (2011). Small animal imaging: basics and practical guide.

[CR32] Kim Y, Venkataraju K, Pradhan K, Mende C, Taranda J, Turaga S, Arganda-Carreras I, Ng L, Hawrylycz M, Rockland K, Seung H, Osten P (2015). Mapping social behavior-induced brain activation at cellular resolution in the mouse. Cell Reports.

[CR33] Klein A, Andersson J, Ardekani BA, Ashburner J, Avants B, Chiang M-C, Christensen GE, Collins DL, Gee J, Hellier P, Song JH, Jenkinson M, Lepage C, Rueckert D, Thompson P, Vercauteren T, Woods RP, Mann JJ, Parsey RV (2009). Evaluation of 14 nonlinear deformation algorithms applied to human brain MRI registration. NeuroImage.

[CR34] Klein S, Staring M, Murphy K, Viergever MA, Pluim JPW (2010). Elastix: a toolbox for intensity-based medical image registration. IEEE Transactions on Medical Imaging.

[CR35] Kuan L, Li Y, Lau C, Feng D, Bernard A, Sunkin SM, Zeng H, Dang C, Hawrylycz M, Ng L (2015). Neuroinformatics of the Allen mouse brain connectivity atlas. Methods.

[CR36] Kumazawa-Manita N, Katayama M, Hashikawa T, Iriki A (2013). Three-dimensional reconstruction of brain structures of the rodent Octodon degus: a brain atlas constructed by combining histological and magnetic resonance images. Experimental Brain Research.

[CR37] Lebenberg J, Hérard AS, Dubois A, Dhenain M, Hantraye P, Delzescaux T (2011). A combination of atlas-based and voxel-wise approaches to analyze metabolic changes in autoradiographic data from Alzheimer’s mice. NeuroImage.

[CR38] Leergaard TB, Hilgetag CC, Sporns O (2012). Mapping the connectome: multi-level analysis of brain connectivity. Frontiers in Neuroinformatics.

[CR39] Lehmann G, Pincus Z, Regrain B (2006). WrapITK: Enhanced languages support for the Insight Toolkit. The Insight Journal.

[CR40] Lein ES, Hawrylycz MJ, Ao N, Ayres M, Bensinger A, Bernard A, Boe AF, Boguski MS, Brockway KS, Byrnes EJ, Chen L, Chen T-M, Chin MC, Chong J, Crook BE, Czaplinska A, Dang CN, Datta S, Dee NR, Desaki AL, Desta T, Diep E, Dolbeare TA, Donelan MJ, Dong H-W, Dougherty JG, Duncan BJ, Ebbert AJ, Eichele G, Estin LK, Faber C, Facer BA, Fields R, Fischer SR, Fliss TP, Frensley C, Gates SN, Glattfelder KJ, Halverson KR, Hart MR, Hohmann JG, Howell MP, Jeung DP, Johnson RA, Karr PT, Kawal R, Kidney JM, Knapik RH, Kuan CL, Lake JH, Laramee AR, Larsen KD, Lau C, Lemon TA, Liang AJ, Liu Y, Luong LT, Michaels J, Morgan JJ, Morgan RJ, Mortrud MT, Mosqueda NF, Ng LL, Ng R, Orta GJ, Overly CC, Pak TH, Parry SE, Pathak SD, Pearson OC, Puchalski RB, Riley ZL, Rockett HR, Rowland SA, Royall JJ, Ruiz MJ, Sarno NR, Schaffnit K, Shapovalova NV, Sivisay T, Slaughterbeck CR, Smith SC, Smith KA, Smith BI, Sodt AJ, Stewart NN, Stumpf K-R, Sunkin SM, Sutram M, Tam A, Teemer CD, Thaller C, Thompson CL, Varnam LR, Visel A, Whitlock RM, Wohnoutka PE, Wolkey CK, Wong VY, Wood M, Yaylaoglu MB, Young RC, Youngstrom BL, Yuan XF, Zhang B, Zwingman TA, Jones AR (2007). Genome-wide atlas of gene expression in the adult mouse brain. Nature.

[CR41] Lerch JP, Yiu AP, Martinez-Canabal A, Pekar T, Bohbot VD, Frankland PW, Henkelman RM, Josselyn SA, Sled JG (2011). Maze training in mice induces MRI-detectable brain shape changes specific to the type of learning. NeuroImage.

[CR42] Ma Y, Hof PR, Grant SC, Blackband SJ, Bennett R, Slatest L, McGuigan MD, Benveniste H (2005). A three-dimensional digital atlas database of the adult C57BL/6J mouse brain by magnetic resonance microscopy. Neuroscience.

[CR43] MacKenzie-Graham A, Lee E-F, Dinov ID, Bota M, Shattuck DW, Ruffins S, Yuan H, Konstantinidis F, Pitiot A, Ding Y, Hu G, Jacobs RE, Toga AW (2004). A multimodal, multidimensional atlas of the C57BL/6J mouse brain. Journal of Anatomy.

[CR44] Mailly P, Haber SN, Groenewegen HJ, Deniau J-M (2010). A 3D multi-modal and multi-dimensional digital brain model as a framework for data sharing. Journal of Neuroscience Methods.

[CR45] Majka, P. (2014). *Integracja danych z obrazowania struktury mózgu oposa krótkoogonowego*. Ph.D. thesis, Nencki Institute of Experimental Biology.

[CR46] Majka, P., Chaplin, T., Yu, H.-H., Pinskiy, V., Mitra, P., Rosa, M., & Wójcik, D.K. (2014). Automated workflow for mapping tracer injection studies of the common marmoset into a reference template. *Frontiers in Neuroinformatics*, *8*(38).

[CR47] Majka P, Kowalski JM, Chlodzinska N, Wójcik DK (2013). 3D Brain Atlas Reconstructor Service-Online Repository of Three-Dimensional Models of Brain Structures. Neuroinformatics.

[CR48] Malandain G, Bardinet E, Nelissen K, Vanduffel W (2004). Fusion of autoradiographs with an MR volume using 2-D and 3-D linear transformations. NeuroImage.

[CR49] Mattes, D., Haynor, D.R., Vesselle, H., Lewellyn, T.K., & Eubank, W. (2001). Nonrigid multimodality image registration. In *SPIE 4322, Medical Imaging 2001: Image Processing*, (Vol. 4322 pp. 1609–1620).

[CR50] Mikula S, Trotts I, Stone JM, Jones EG (2007). Internet-enabled high-resolution brain mapping and virtual microscopy. NeuroImage.

[CR51] Morris JA, Royall JJ, Bertagnolli D, Boe AF, Burnell JJ, Byrnes EJ, Copeland C, Desta T, Fischer SR, Goldy J, Glattfelder KJ, Kidney JM, Lemon T, Orta GJ, Parry SE, Pathak SD, Pearson OC, Reding M, Shapouri S, Smith KA, Soden C, Solan BM, Weller J, Takahashi JS, Overly CC, Lein ES, Hawrylycz MJ, Hohmann JG, Jones AR (2010). Divergent and nonuniform gene expression patterns in mouse brain. Proceedings of the National Academy of Sciences of the United States of America.

[CR52] Nikou C (2003). A robust statistics-based global energy function for the alignment of serially acquired autoradiographic sections. Journal of Neuroscience Methods.

[CR53] Oh SW, Harris JA, Ng L, Winslow B, Cain N, Mihalas S, Wang Q, Lau C, Kuan L, Henry AM, Mortrud MT, Ouellette B, Nguyen TN, Sorensen SA, Slaughterbeck CR, Wakeman W, Li Y, Feng D, Ho A, Nicholas E, Hirokawa KE, Bohn P, Joines KM, Peng H, Hawrylycz MJ, Phillips JW, Hohmann JG, Wohnoutka P, Gerfen CR, Koch C, Bernard A, Dang C, Jones AR, Zeng H (2014). A mesoscale connectome of the mouse brain. Nature.

[CR54] Osten P, Margrie TW (2013). Mapping brain circuitry with a light microscope. Nature Methods.

[CR55] Palm C, Axer M, Gräßel D, Dammers J, Lindemeyer J, Zilles K, Pietrzyk U, Amunts K (2010). Towards ultra-high resolution fibre tract mapping of the human brain - registration of polarised light images and reorientation of fibre vectors. Frontiers in Human Neuroscience.

[CR56] Papp EA, Leergaard TB, Calabrese E, Johnson GA, Bjaalie JG (2014). NeuroImage Waxholm Space atlas of the Sprague Dawley rat brain. NeuroImage.

[CR57] Peng H, Chung P, Long F, Qu L, Jenett A, Seeds AM, Myers EW, Simpson JH (2011). BrainAligner: 3D registration atlases of Drosophila brains. Nature Methods.

[CR58] Pitiot A, Bardinet E, Thompson PM, Malandain G (2006). Piecewise affine registration of biological images for volume reconstruction. Medical Image Analysis.

[CR59] Pitiot A, Guimond A (2008). Geometrical regularization of displacement fields for histological image registration. Medical Image Analysis.

[CR60] Poline J-B, Breeze JL, Ghosh S, Gorgolewski K, Halchenko YO, Hanke M, Haselgrove C, Helmer KG, Keator DB, Marcus DS, Poldrack RA, Schwartz Y, Ashburner J, Kennedy DN (2012). Data sharing in neuroimaging research. Frontiers in Neuroinformatics.

[CR61] Qiu X, Shi L, Pridmore T, Pitiot A, Wang D (2011). Atlas-guided correction of brain histology distortion. Journal of Pathology Informatics.

[CR62] Ragan T, Kadiri LR, Venkataraju KU, Bahlmann K, Sutin J, Taranda J, Arganda-Carreras I, Kim Y, Seung HS, Osten P (2012). Serial two-photon tomography for automated ex vivo mouse brain imaging. Nature Methods.

[CR63] Ribes, D., Parafita, J., Charrier, R., Magara, F., Magistretti, P.J., & Thiran, J.P. (2010). JULIDE: a software tool for 3D reconstruction and statistical analysis of autoradiographic mouse brain sections. *PLoS ONE*, *5* (11). 10.1371/journal.pone.0014094 PMC2991313 21124830

[CR64] Rosa MGP, Palmer SM, Gamberini M, Burman KJ, Yu H-H, Reser DH, Bourne JA, Tweedale R, Galletti C (2009). Connections of the dorsomedial visual area: pathways for early integration of dorsal and ventral streams in extrastriate cortex. The Journal of Neuroscience: the Official Journal of the Society for Neuroscience.

[CR65] Sawiak SJ, Wood NI, Williams GB, Morton AJ, Carpenter TA (2009). Use of magnetic resonance imaging for anatomical phenotyping of the R6/2 mouse model of Huntington’s disease. Neurobiology of Disease.

[CR66] Schroeder, W. (2005). The ITK Software Guide Second Edition Updated for ITK version 2. 4.

[CR67] Sukhinin, D.I., Engel, A.K., Manger, P., & Hilgetag, C.C. (2015). Building the Ferretome. pages 1–14. 10.3389/fninf.2016.00016 PMC4861729 27242503

[CR68] Tange O (2011). GNU Parallel: The Command-Line Power Tool. Login the USENIX Magazine.

[CR69] Thévenaz P, Ruttimann UE, Unser M (1998). A pyramid approach to subpixel registration based on intensity. IEEE Transactions on Image Processing.

[CR70] Tustison NJ, Johnson HJ, Rohlfing T, Klein A, Ghosh SS, Ibanez L, Avants BB (2013). Instrumentation bias in the use and evaluation of scientific software: recommendations for reproducible practices in the computational sciences. Frontiers in Neuroscience.

[CR71] Uberti M, Liu Y, Dou H, Mosley RL, Gendelman HE, Boska M (2009). Registration of in vivo MR to histology of rodent brains using blockface imaging. Proceedings of SPIE.

[CR72] Ullmann JF, Janke AL, Reutens D, Watson C (2015). Development of MRI-based atlases of non-human brains. Journal of Comparative Neurology.

[CR73] Ullmann JFP, Keller MD, Watson C, Janke AL, Kurniawan ND, Yang Z, Richards K, Paxinos G, Egan GF, Petrou S, Bartlett P, Galloway GJ, Reutens DC (2012). Segmentation of the C57BL/6J mouse cerebellum in magnetic resonance images. NeuroImage.

[CR74] Veraart J, Leergaard TB, Antonsen BRT, Van Hecke W, Blockx I, Jeurissen B, Jiang Y, Van der Linden A, Johnson GA, Verhoye M, Sijbers J (2011). Population-averaged diffusion tensor imaging atlas of the Sprague Dawley rat brain. NeuroImage.

[CR75] Vousden, D.A., Epp, J., Okuno, H., Nieman, B.J., van Eede, M., Dazai, J., Ragan, T., Bito, H., Frankland, P.W., Lerch, J.P., & Henkelman, R.M. (2014). Whole-brain mapping of behaviourally induced neural activation in mice. *Brain Structure and Function*, 1–15. 10.1007/s00429-014-0774-0 24760545

[CR76] Wang C-W, Budiman Gosno E, Li Y-S (2015). Fully automatic and robust 3D registration of serial-section microscopic images. Scientific Reports.

[CR77] Wang C-W, Ka S-M, Chen A (2014). Robust image registration of biological microscopic images. Scientific Reports.

[CR78] Yang Z, Richards K, Kurniawan ND, Petrou S, Reutens DC (2012). MRI-guided volume reconstruction of mouse brain from histological sections. Journal of Neuroscience Methods.

[CR79] Yelnik J, Bardinet E, Dormont D, Malandain G, Ourselin S, Tandé D, Karachi C, Ayache N, Cornu P, Agid Y (2007). A three-dimensional, histological and deformable atlas of the human basal ganglia. I. Atlas construction based on immunohistochemical and MRI data. NeuroImage.

[CR80] Yushkevich PA, Avants BB, Ng L, Hawrylycz M, Burstein PD, Zhang H, Gee JC, Pluim JPW, Likar B, Gerritsen FA (2006). 3D mouse brain reconstruction from histology using a coarse-to-fine approach. Lecture Notes in Computer Science (including subseries Lecture Notes in Artificial Intelligence and Lecture Notes in Bioinformatics), volume 4057 LNCS of Lecture Notes in Computer Science.

[CR81] Yushkevich PA, Piven J, Hazlett HC, Smith RG, Ho S, Gee JC, Gerig G (2006). User-guided 3D active contour segmentation of anatomical structures: significantly improved efficiency and reliability. NeuroImage.

[CR82] Zaborszky L, Csordas A, Mosca K, Kim J, Gielow MR, Vadasz C, Nadasdy Z (2015). Neurons in the basal forebrain project to the cortex in a complex topographic organization that reflects corticocortical connectivity patterns: an experimental study based on retrograde tracing and 3D reconstruction. Cerebral Cortex.

[CR83] Zakiewicz IM, Majka P, Wójcik DK, Bjaalie JG, Leergaard TB (2015). Three-dimensional histology volume reconstruction of axonal tract tracing data: exploring topographical organization in subcortical projections from rat barrel cortex. PLOS ONE.

[CR84] Zilles K (1985). The Cortex of the Rat.

